# Exploration of anti-insect potential of trypsin inhibitor purified from seeds of *Sapindus mukorossi* against *Bactrocera cucurbitae*

**DOI:** 10.1038/s41598-019-53495-6

**Published:** 2019-11-19

**Authors:** Samiksha, Drishtant Singh, Anup Kumar Kesavan, Satwinder Kaur Sohal

**Affiliations:** 1 Department of Zoology, Guru Nanak Dev University Amritsar, Punjab, 143005 India; 2 Department of Molecular Biology and Biochemistry, Guru Nanak Dev University Amritsar, Punjab, 143005 India

**Keywords:** Plant sciences, Zoology

## Abstract

Peptidase inhibitors (PIs) are defense proteins of plants which are active against gut peptidases of different insects. *Sapindus mukorossi* was identified as a source of bioactive PIs which could confer resistance against *Bactrocera cucurbitae*, a most devastating pest of several economically important crops. In the present study, a trypsin inhibitor was purified from mature dry seeds of *S. mukorossi* and characterized for its biochemical properties as well as its potential for bio control of *B. cucurbitae*. The purified fractions from RP- HPLC through SDS-PAGE gave an apparent molecular weight of ~29 kDa. *S. mukorossi* trypsin inhibitor (SMTI) was found to be a non-competitive inhibitor which was active over a broad range of temperature (10–100 °C) and pH (6–11). SMTI when incorporated in artificial diet inhibited the growth and development of *B. cucurbitae* larvae. Gene expression analysis of trypsin and chymotrypsin genes via qRT-PCR indicated that their mRNA expression was down-regulated while that of other genes namely, Catalase, Elastase, Superoxide Dismutase, Glutathione –S-transferase and Alkaline Phosphatase was up regulated. SMTI also showed deleterious effects against different bacterial strains. The results of this study indicated that *S. mukorossi* trypsin inhibitor has potential to be used as a bio control agent that can reduce the harm caused by melon fruit fly and other devastating pests.

## Introduction

The losses caused by insect pest attack on economically important crops have reached upto 20% in large cultivars and have become a matter of serious concern in food production^1^. The peptidase inhibitors (PIs) are a class of plant proteins that play an important role in natural defense mechanism provoked by herbivory^2^ and can be explored for lessen the destruction caused by pathogens and insects. Plant peptidase inhibitors (PPIs) are widely distributed in different plant families like leguminaceae, sapoteaceae and exist in various plant parts like flowers, leaves, stem including seeds, where these comprise upto 10% of total plant proteins^3^. Serine PIs are the most represented family of these plant PIs which include Kunitz and Bowman-Birk inhibitors having molecular mass ranging between 18 and 22 kDa. These typically inhibit chymotrypsin and trypsin peptidases and generally form two disulfide bridges with four cysteine residues^4,5^. Transgenic plants exhibiting PIs expression have been evaluated as an alternative approach for defense against insect pests as they are safe and environment friendly compared to chemical pesticides^6^. Therefore, there is a need to explore India’s large plant diversity for PIs which can be exploited for insect pest management.

*Bactrocera cucurbitae* is a polyphagous pest that attacks over 81 species of plants^7^. It is a significant economic pest of cucurbit crops, viz., *Momordica charantia* L. (*Cucumis sativus* L.), *Cucumis anguria* L., *Trichosanthes anguina* L., *Luffa acutangula* Roxb., *Citrullus lanatus* L., *Cucurbita moschata* Duchesne, *Cucumis melo* L. etc. Depending upon the season and cucurbit species, the extent of losses caused by melon fruit fly ranges from 30 to 100%^8^. *Sapindus mukorossi* is a deciduous tree belonging to the family Sapindaceae, also known as reetha, commonly found in North India. *S. mukorossi* has been extensively explored for its pharmacological action which include anti inflammatory, antimicrobial and hepato-protective activity. The ethanolic extracts of the plant have also been explored for insecticidal activity against *Sitophilus oryzae* and *Pediculus humanus* where the extracts caused significant mortality and repellent effect on the insects^9^. However, no studies have been carried out to evaluate the effect of the plant PIs from *S. mukorossi* against economically important agricultural insect pests. Therefore, in present study a trypsin inhibitor from *S. mukorossi* seeds was purified, characterized and further evaluated for its insecticidal effects against *B. cucurbitae* using bioassays, enzyme assays and gene expression analyses.

## Results

### Purification of SMTI

**Figure 1:**
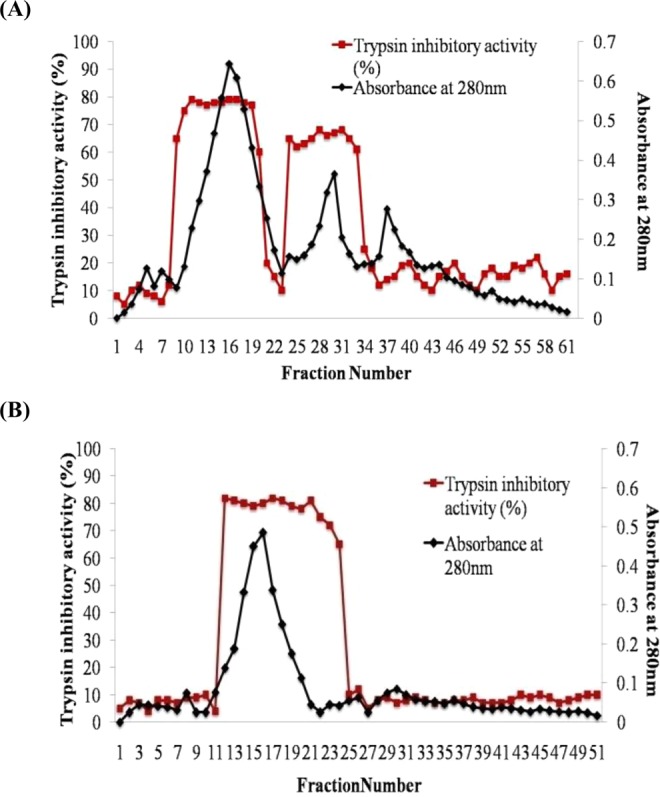
Purification profile of SMTI. Elution profile of (**A**) DEAE-cellulose column loaded with partially purified fraction obtained after dialysis; (**B**) Trypsin- Sepharose 6B column loaded with fraction (No. 7–22) pooled from the DEAE-cellulose column.

**Figure 2:**
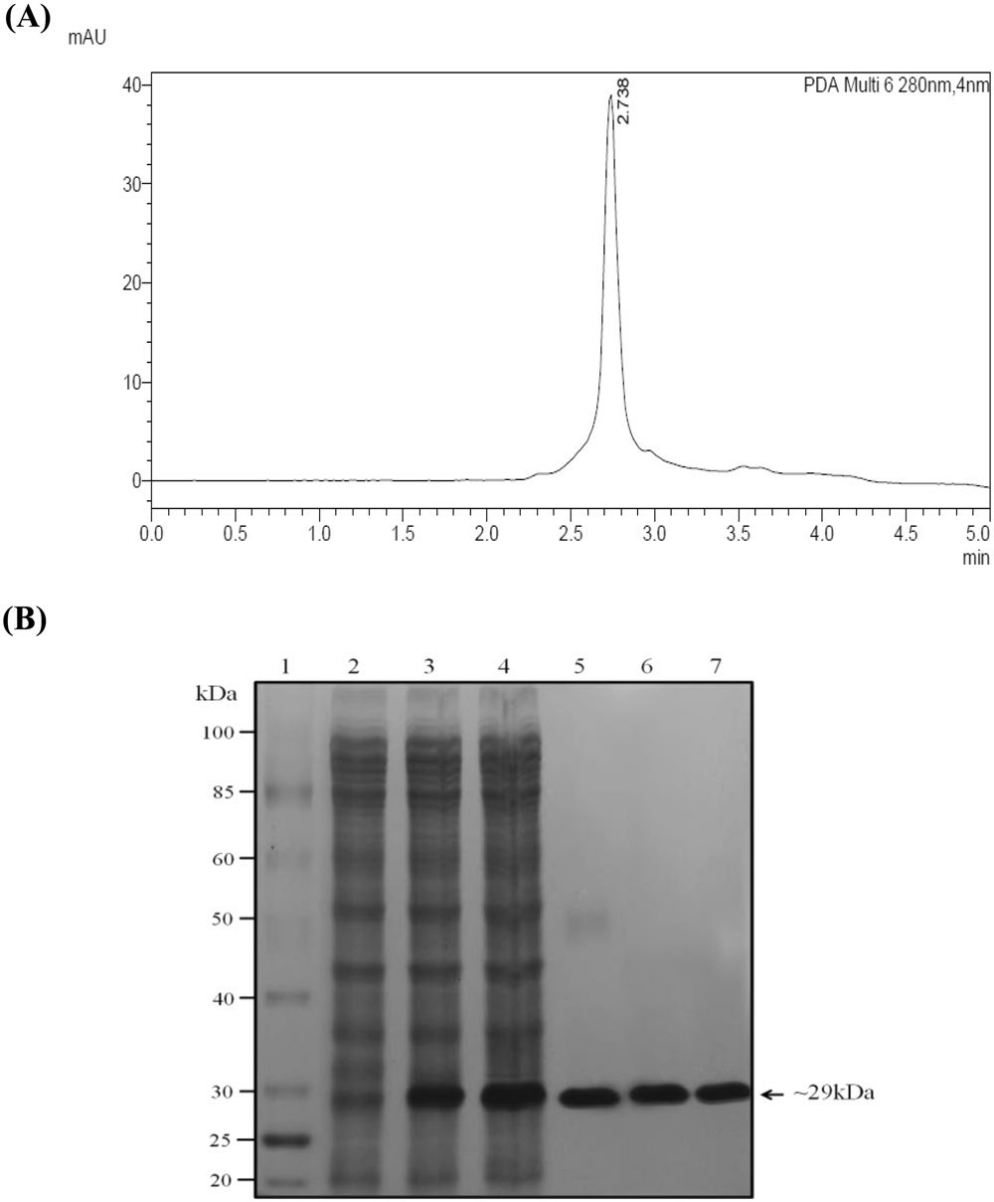
(**A**) RP-HPLC chromatogram obtained after injection into C-18G column showing single peak at a retention time of 2.738 min (**B**) SDS-PAGE gel showing different protein fractions during each purification step. Lane- 1 benchmark prestained protein ladder (Invitrogen), lane-2 Crude extract, lane-3 ammonium sulphate precipitated fraction (0–80%), lane-4 partially purified fraction, lane-5 DEAE-cellulose fraction, lane-6 trypsin sepharose 6B fraction, lane-7 RP-HPLC purified fractions.

Trypsin inhibitor from *S. mukorossi* seeds was purified using ammonium sulphate precipitation followed by dialysis. It was found that 0–80% (F1) saturated protein fraction gave the maximum trypsin inhibitory activity compared to 20–80% (F2), 40–80% (F3), and 60–80% (F4) saturated fractions. SMTI was further purified using different chromatographic techniques. In the first step, DEAE cellulose chromatography separated the partially purified fractions into two peaks which showed maximum inhibitory activity for trypsin (Fig. 1A). With DEAE column, the trypsin inhibitor was purified to 3.46 fold with 66.85% yield and specific activity of 295 TIUmg^−1^ protein (Table 1). In the second step, peak showing the maximum trypsin inhibitory activity was subjected to trypsin sepharose 6B affinity chromatography and the eluted peak (Fig. 1B) exhibited highest trypsin inhibitory activity with specific activity 694 TIUmg^−1^ and 8.14 purification fold. The fractions/eluent from the affinity chromatography column was subjected to reverse phase HPLC which gave a single peak with a retention time of 2.738 min indicating the presence of single purified inhibitor (Fig. 2A). RP-HPLC gave 9.26 fold purified trypsin inhibitor with a yield of 10.47% and specific activity of 789 TIUmg^−1^ protein (Table 1).

**Table 1:** Purification steps of SMTI.

Steps	Total protein^a^	Total TIU^b^	Specific activity (TIU mg^−1^ protein)	Yield^c^ (%)	Purification index^d^
Crude Extract (CE)	163250	13917062.5	85.25	100	1
Partially Purified	67760	8537760	126	61.34	1.48
Ion Exchange	31539.2	9304064	295	66.85	3.46
Affinity	9028.8	6265987.2	694	45.02	8.14
RP-HPLC	1846.99	1457274.32	789	10.47	9.26

^a^Total amount of protein extracted from 100 g of defatted flour from *S. mukorossi* seeds.

^b^One TIU was defined as the decrease in 0.01 unit of absorbance at 405 nm.

^c^Recovery at each purification step (Crude extract, 100%).

^d^Purification index is calculated as the ratio between the specific activity obtained at each purification step and that of the CE taken as 1.0.

### Characterization of SMTI

#### SDS PAGE

12% SDS-PAGE showed that SMTI exhibited a molecular mass of ~29 kDa, indicating that the inhibitor obtained from affinity chromatography and RP-HPLC column contains a single polypeptide chain (Fig. 2B).

#### Effect of temperature and pH

**Figure 3:**
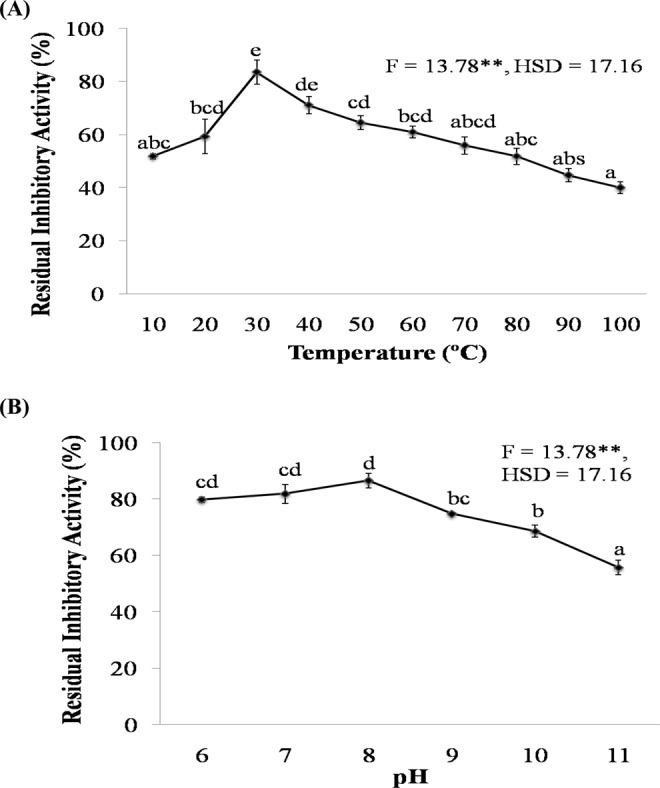
(**A**) Temperature stability of SMTI after 30 min incubation at varying temperatures. (**B**) pH stability of SMTI after incubation at varying pH for 30 min at 25 °C. Data are mean ± Standard Deviation, two way ANOVA and Tukey’s HSD. Treatments with same letter indicate no significant difference p < 0.01. ^*^And ^**^ indicates significant at p < 0.01 and p < 0.001, respectively. F = F-ratio; HSD = Honestly Significance Difference.

The inhibitory activity of SMTI increased with increase in temperature i.e. 10–30 °C, after that it declined but retained ~40% of the inhibitory activity at 100 °C after incubation of 30 min. Maximum trypsin inhibitory activity (83%) was observed at 30 °C (Fig. 3A). SMTI exhibits stability at broad pH range (pH 6.0–11.0). The maximum activity (~87%) was observed at pH 8.0. The SMTI was active in both acidic and basic range. It retained 56% of inhibitory activity at pH-11.0 (Fig. 3B).

#### Effect of metal ions, detergents and organic solvents

**Figure 4:**
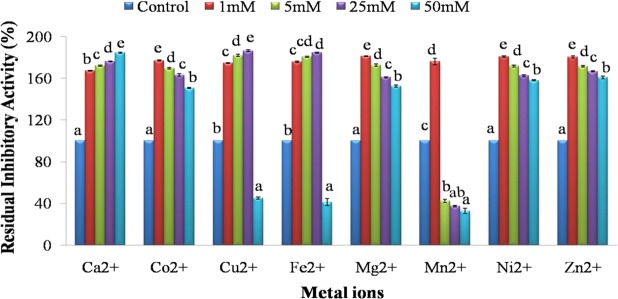
Metal ions stability of SMTI after 30 min incubation at room temperature. Data are mean ± Standard Deviation, two way ANOVA and Tukey’s HSD. Treatments with same letter indicate no significant difference p < 0.01. Ca^2+^(F = 6410.16**, HSD = 1.97), Co^2+^(F = 1479.83**, HSD = 3.7), Cu^2+^(F = 4472.91**, HSD = 4.35), Fe^2+^ (F = 1715.8^2**^, HSD = 7.13), Mg^2+^ (F = 1713.9^2**^, HSD = 3.57), Mn^2+^ (F = 999.78**, HSD = 9.02), Ni^2+^ (F = 1854.06**, HSD = 3.43) and Zn^2+^ (F = 1255.18**, HSD = 4.2). ^*^And ^**^ indicates significant at p < 0.01 and p < 0.001, respectively. F = F-ratio; HSD = Honestly Significance Difference.

Trypsin inhibitory activity of SMTI increased with increase in concentration of Ca^2+^, Co^2+^, Mg^2+^, Zn^2+^, Ni^2+^ which ranged from 1 mM to 50 mM and for Cu^2+^ and Fe^2+^ increased from 1 mM to 25 mM but reduced at 50 mM. However it increased at 1 mM but reduced at 5 mM, 25 mM and 50 mM for Mn^2+^ (Fig. 4).

**Figure 5:**
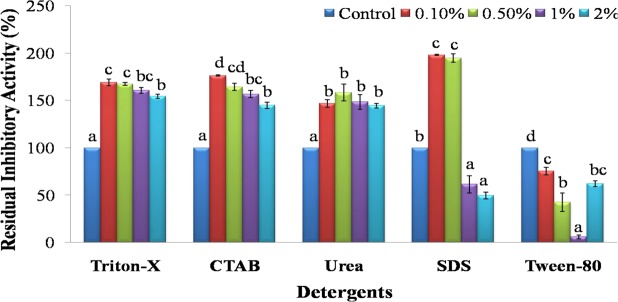
Detergent stability of SMTI after 30 min incubation at room temperature. Data are mean ± Standard Deviation, two way ANOVA and Tukey’s HSD. Treatments with same letter indicate no significant difference p < 0.01. Triton X (F = 151.47, HSD = 10.84), CTAB (F = 102.94**, HSD = 13.45), Urea (F = 15.8, HSD = 26.53), SDS (F = 218.9**, HSD = 22.48), Tween 80 (F = 50.85, HSD = 23.12). ^*^And ^**^ indicates significant at p < 0.01 and p < 0.001, respectively. F = F-ratio; HSD = Honestly Significance Difference.

Trypsin inhibitory activity of SMTI decreased upon addition of Tween-80. It was also decreased in the presence of other detergents like Triton-X, CTAB and Urea. But, there was a slight increase in the presence of SDS upto 5 mM there after it was reduced (Fig. 5).

**Figure 6:**
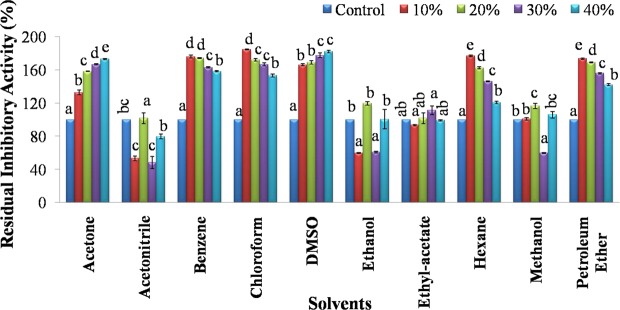
Solvents stability of SMTI after 30 min incubation at room temperature. Data are mean ± Standard Deviation, two way ANOVA and Tukey’s HSD. Treatments with same letter indicate no significant difference p < 0.01. Acetone (F = 531.86**, HSD = 6.06), Acetonitrile (F = 29.03**, HSD = 21.74), Benzene (F = 1210.05**, HSD = 4.19), Chloroform (F = 507.74**, HSD = 6.81), DMSO (F = 354.81**, HSD = 8.31), Ethanol (F = 25.17**, HSD = 24.78), Ethyl-acetate (F = 3.1, HSD = 17.29), Hexane (F = 971.39**, HSD = 4.64), Methanol (F = 86.37**, HSD = 10.81), Petroleum Ether (F = 1484.5**, HSD = 3.58). ^*^And ^**^ indicates significant at p < 0.01 and p < 0.001, respectively. F = F-ratio; HSD = Honestly Significance Difference.

SMTI activity increased slightly at 10%, 20%, 30% and 40% concentrations of acetone, benzene, chloroform, DMSO, hexane and petroleum ether (Fig. 6). No significant effect of ethyl acetate, acetonitrile and methanol was noticed on its activity.

#### IC_50_, IC_90,_ Casein hydrolysis test and Kinetic Study

**Figure 7:**
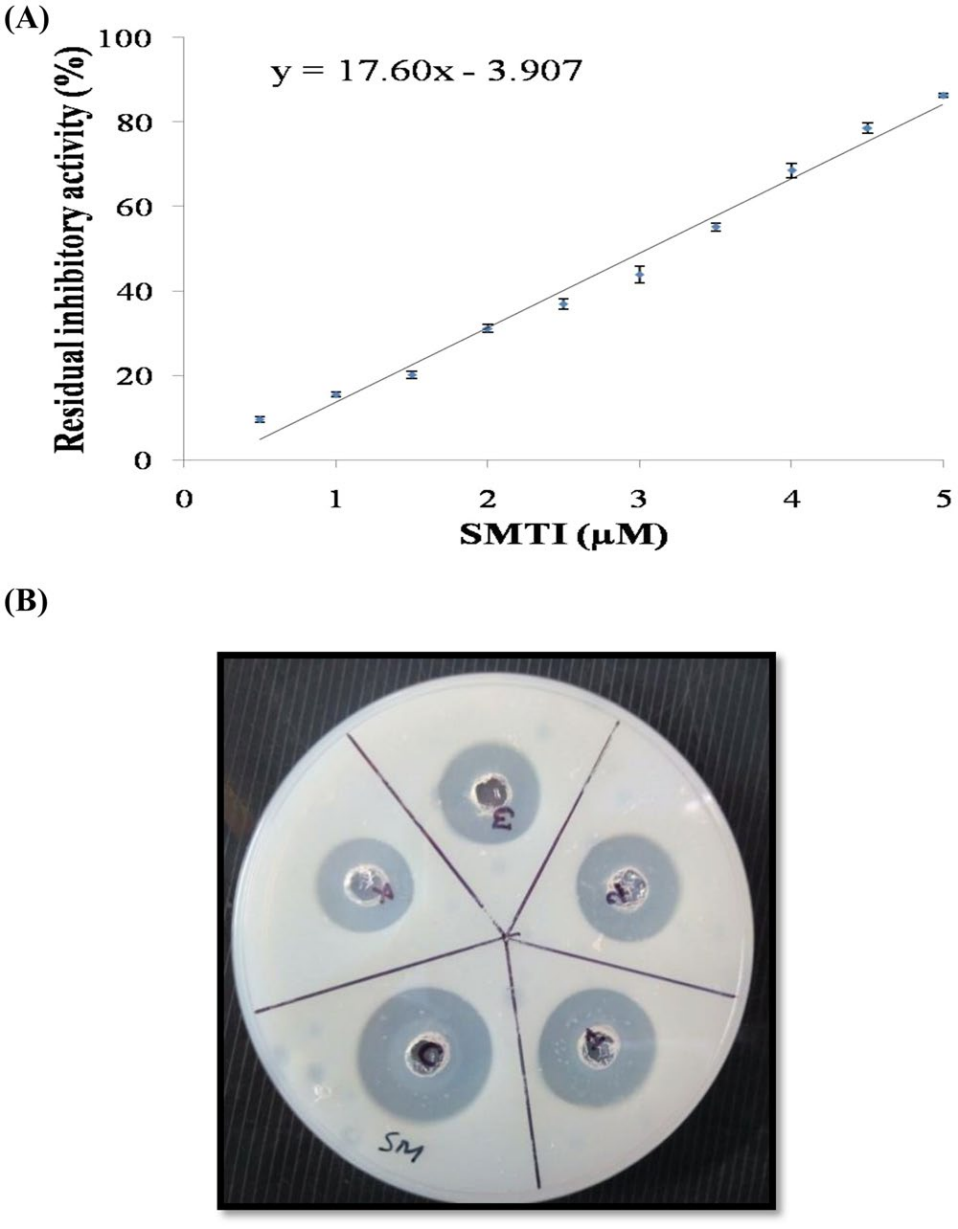
(**A**) Effect of SMTI against bovine trypsin. The IC_50_ of SMTI on trypsin was 3.07 ± 0.08 µM and IC_90_ was 5.34 ± 0.14 µM. (**B**) Casein hydrolysis test: Zone of inhibition caused by different concentrations of SMTI in wells 1, 2, 3, 4. Well 1 = 25 µg/ml, Well 2 = 50 µg/ml, Well 3 = 75 µg/ml, Well 4 = 100 µg/ml, C = Control (Without inhibitor).

**Figure 8:**
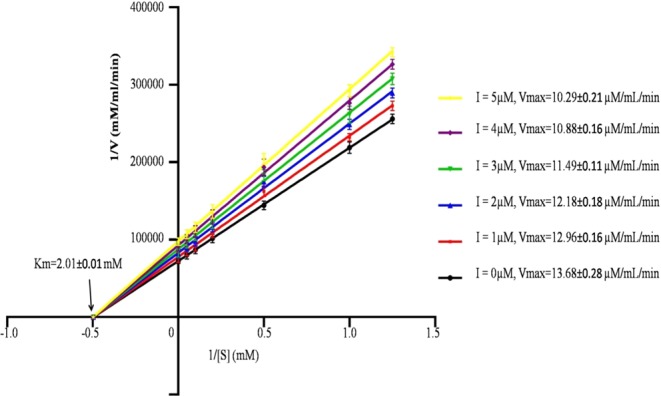
Inhibition kinetics of SMTI: Kinetic analysis of trypsin using SMTI as an enzyme inhibitor and BApNA as a substrate. The mechanism of inhibition was evaluated using Line weaver Burk plot (or double reciprocal lot), in which the inverse of the initial rate was plotted against the inverse of the substrate concentrations representing the inhibitory effect of the SMTI in presence of its different concentrations from 1.0 μM to 5.0 μM on trypsin.

The IC_50_ and IC_90_ values of SMTI obtained from linear regression equation (Y = 17.60 × 3.907) were 3.07 ± 0.08 µM and 5.34 ± 0.14 µM, respectively (Fig. 7A). The inhibition kinetics of SMTI was evaluated using Line weaver Burk plot. In the presence of inhibitor there was a decrease in V_max_ but there was no significant change in Km which suggested that the mode of inhibition of trypsin by SMTI was non-competitive (Fig. 8). The Ki value obtained for SMTI was 15.73 ± 0.16 µM.

The hydrolysis zone around the wells decreased with increase in concentration of SMTI as compared to control (Fig. 7B) indicating trypsin inhibition by SMTI (Table 2). The least hydrolysis zone was observed at highest concentration i.e. at 100 µg/ml in well 4.

**Table 2:** Zone of inhibition formed by different concentrations of SMTI in agar plates.

S. No.	Concentration of SMTI (µg/ml)	Zone of inhibition (mm*)
1	0	15
2	25	11
3	50	8
4	75	5
5	100	3

*The values are mean of triplicates.

#### Larval Growth Index (LGI) and Total Growth Index (TGI)

**Figure 9:**
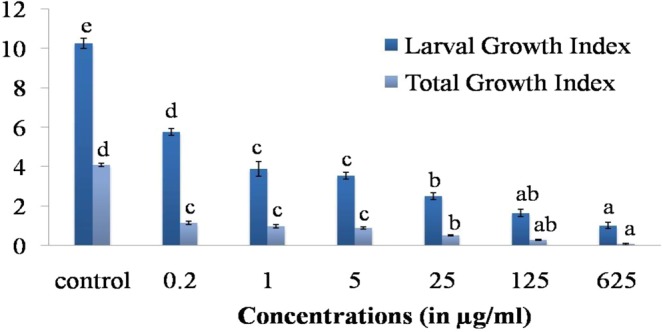
Larval Growth Index (LGI) and Total Growth Index (TGI) of *B. cucurbitae* in days when second instar larvae were fed on different concentrations SMTI. Data are mean ± Standard Deviation, two ways ANOVA and Tukey’s HSD (six biological replicates). Treatments with same letter indicate no significant difference p < 0.01. LGI (F = 186.89^**^, HSD = 0.98), TGI (F = 416.32^**^, HSD = 0.28). ^*^And ^**^ indicates significant at p < 0.01 and p < 0.001, respectively. F = F-ratio; HSD = Honestly Significance Difference.

Growth indices results revealed that the LGI and TGI of second instar larvae of *B. cucurbitae* was significantly (F_6,35_ = 186.89; 416.32 P < 0.001) reduced when fed with different concentrations of SMTI incorporated in artificial diet. At 625 µg/ml, LGI and TGI were markedly reduced by 90.14% and 92.89% respectively as compared to the control (Fig. 9).

### Enzyme assay

**Figure 10:**
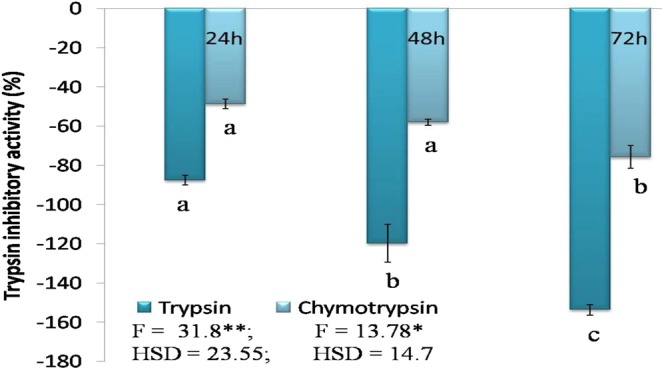
Effect of LC_50_ (6.98 µg/ml) of SMTI on Trypsin and Chymotrypsin enzymes of second instar larvae of *B. cucurbitae* at 24 h, 48 h and 72 h. Data are mean ± Standard Deviation, two way ANOVA and Tukey’s HSD (six biological replicates). Treatments with same letter indicate no significant difference p < 0.01. ^*^And ^**^ indicates significant at p < 0.01 and p < 0.001, respectively. F = F-ratio; HSD = Honestly Significance Difference.

Inhibitory effects of SMTI at LC_50_ concentration (6.98 µg/ml) (Fig. S1) were observed for both trypsin and chymotrypsin in gut extracts. The activity of trypsin and chymotrypsin decreased significantly in all the time intervals i.e. at 24, 48 and 72 h when second instar larvae (64–72 h) were fed with LC_50_ concentration of SMTI. The decrease in activity of both enzymes was found to increase with increase in treatment interval (Fig. 10).

### Gene expression analysis

**Figure 11:**
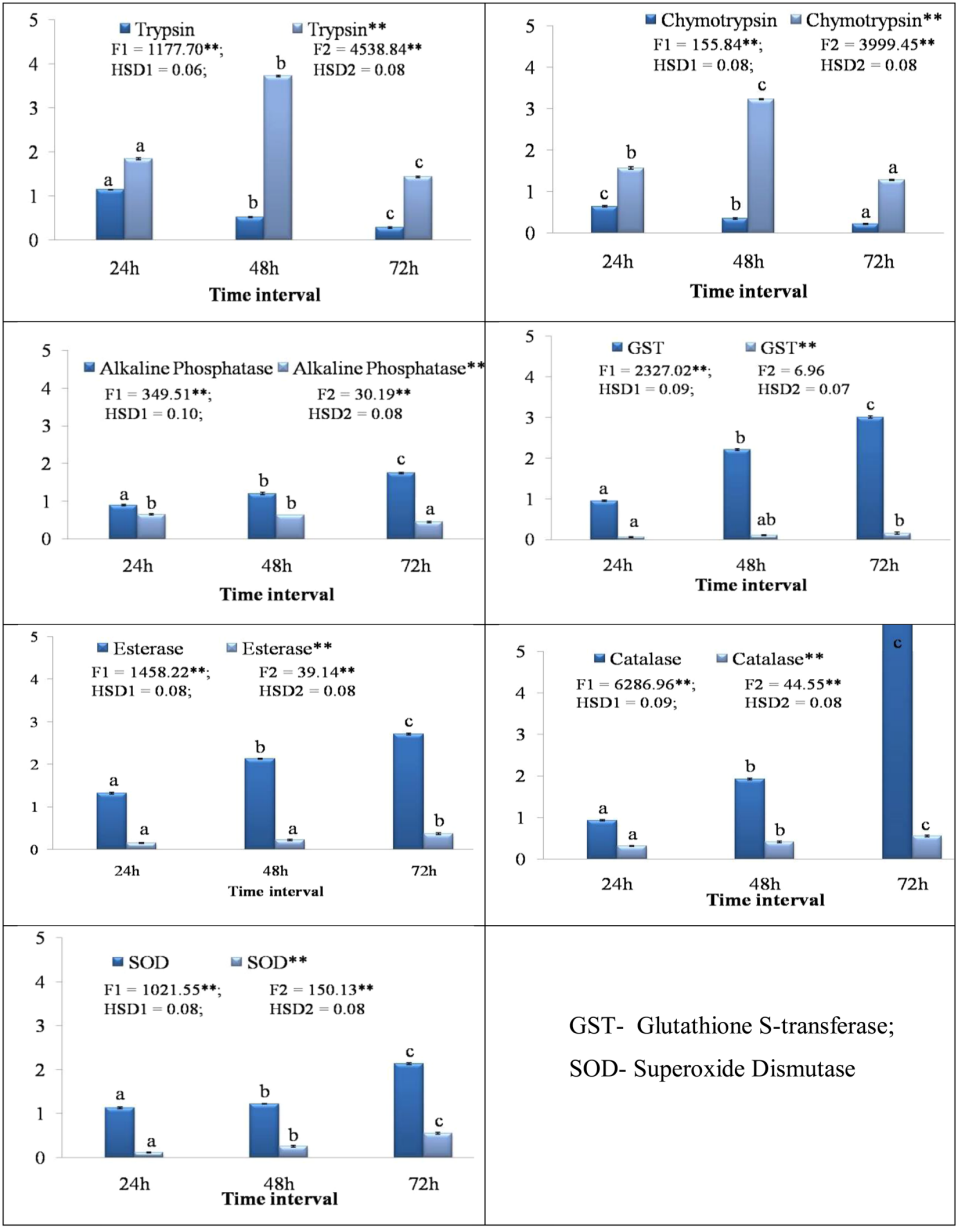
Effect of SMTI on different genes of second instar larvae of *B. cucurbitae* fed on artificial diet supplemented with and without SMTI (**indicates without SMTI). Data are mean ± Standard Deviation, two way ANOVA and Tukey’s HSD. Treatments with same letter indicate no significant difference p < 0.01. ^*^And ^**^ indicates significant at p < 0.01 and p < 0.001, respectively.

The change in expression level of various genes in (64–72 h) larvae (2nd instar) of melon fruit fly fed on LC_50_ concentration (6.98 μg/ml) of SMTI was investigated using qRT-PCR. There was a considerable down regulation of genes involved in digestion (trypsin and chymotrypsin) after 24, 48 and 72 h of incorporating SMTI in the diet. At 72 h, the resultant fold change in mRNA level of trypsin was 0.28 and that of chymotrypsin was 0.22. On the other hand, the mRNA level of esterase in the presence of SMTI was elevated by 2.71 fold as compared to control at 72 h. The mRNA level of SOD, AP, catalase and GST was also up regulated in second instar larvae due to the effect of SMTI in comparison to control (Fig. 11).

#### Antibacterial activity

SMTI was shown to have antibacterial activity against *Bacillus thuringiensis, Escherichia coli* and *Pseudomonas aeruginosa* (Table 3). However, SMTI was found to have no inhibitory effect on *Mycobacterium smegmatis* (Fig. S2).

**Table 3:** Antibacterial activity of SMTI.

Concentration of SMTI (µg/ml)	Radius of zone of inhibition (in mm)			
	*E. coli*	*M. smeg*	*B.t*.	*P.a*.
50	7	0	5	6
100	9	0	7	8
150	11	0	9	10
200	13	0	11	12
Control	A-13	R-13	K-12	C-13

*E.coli-Escherichia coli (DH5α), M.smeg-Mycobacterium smegmatis (Mcc155), B.t.-Bacillus thuringiensis (kurstaki), P.a.-Pseudomonas aeruginosa*, A-Ampicillin, R-Rifampin, K-Kanamycin, C-Carbenicillin.

## Discussion

Plants are constantly exposed to stress due to changing environmental conditions. In addition, pathogens and predators continuously attack them. To overcome these adverse conditions plants produce toxic substances to reduce insect damage^10^. PIs are among these compounds which have attracted significant attention because of their role in plant defense and possible applications in transgenic plants to enhance resistance to pathogens and other insect pests. In view of the insect control potential of PIs, we purified trypsin inhibitor from *S. mukorossi* seeds and assessed its insecticidal effect on *B. cucurbitae* larvae along with its antibacterial effect.

A trypsin inhibitor from *S. mukorossi* seeds (SMTI) was purified with a recovery of 10.47% and purification fold of 9.26 which was greater than the trypsin inhibitor purified from *Inga vera* seeds^11^ (yield 7.14%; purification fold 1.36) and *Lonchocarpus sericeus* seeds^10^ (yield 0.88%; purification fold 8.26). SDS-PAGE depicted a molecular mass of ~29 kDa, similar to that of other trypsin inhibitors^12,13^.

Serine peptidase inhibitors are resistant to extreme pH and temperature and this uncommon stability is due to non-covalent interactions and disulfide bonds in the inhibitor molecule^14^. The SMTI showed stability over wide temperature and pH range which indicates that, SMTI possesses high intrinsic stability in its native state. These characteristic were also noted in other trypsin inhibitors^15–17^. A broad range of temperature and pH were studied to check the stability of peptidase inhibitor with respect to seasonal and daily temperature variation. Also there is so much of variation in pH of soil and different plants/plant parts around different demographic areas. SMTI inhibitory activity was also influenced by different metal ions (Mn^2+^ Co^2+^, Ni^2+^, Fe^3+^, Cu^2+^, Zn^2+^, Ca^2+^, Mg^2+^), solvents (DMSO, benzene, petroleum-ether acetone, hexane, and chloroform) and detergents (SDS, CTAB, Triton X-100, Urea, Tween-80). The activity of peptidase inhibitor purified from the seeds of *Albizia amara* was affected in the presence of different metal ions (increased in presence of Cu^2+^ and decreased in presence of Ni^2+^, Fe^3+^, Ba^2+^, Mn^2+^ and Mg^2+^) and detergents (enhanced in presence of Triton X-100 and diminished in presence of Tween-20, SDS and Tween – 80)^18^. However, the activity of trypsin inhibitor purified from *D. biflorus* declined in presence of Cu^2+^ and Ni^2+^ but DMSO enhanced the chymotrypsin inhibitory activity while no effect was observed on trypsin inhibitory activity^19^. The binding of different metal ions might cause a conformational change in a protein (peptidase inhibitor) which may or may not favour its binding to protein (trypsin), thus resulting in inhibition or no inhibition^20,21^. Metal ions play a vital role in protecting the structural stability of PIs that is essential for different biological actions and is susceptible to oxidative/reductional damage^22,23^. Bacha *et al*.^24^ noted that the cumulative impact of PIs and detergents enhances membrane protein solubilization and other physiological components as well as reduces undesirable proteolysis. In addition, solvents are commonly used with PIs for purification, solubilization, and stabilization^18^. Solvents and detergents increase the solubility of the protein and lower the risk of microbial contamination during preparations/downstream processing, therefore they are tested in the present study^25^.

With regards to mechanism of action, SMTI showed a non-competitive type of inhibition similar to that reported in *Sapindus trifoliatus* seeds^12^. Vogel *et al*.^26^ noted that most inhibitors adopt a inhibition kinetics of non-competitive type, although a few trypsin inhibitors have exhibited a competitive form of inhibition^27–29^. The low value of Ki depicts strong affinity of SMTI to trypsin.

The decrease in growth rate of *B. cucurbitae* larvae indicated by decline in percentage pupation and percentage emergence suggests a deterrent effect of the SMTI on the development of the 2^nd^ instar larvae (64–72 h) of melon fruit fly. Kaur and Sohal^30^, perceived that different concentrations of the pea peptidase inhibitor, when added in artificial diet of second instar larvae (64–72 h) of *B. cucurbitae*, decreased the larval growth and total development period. Results analogous to present findings were also observed by Kaur *et al*.^31^ in *B. cucurbitae* where they found that soybean trypsin-chymotrypsin inhibitor (Bowman–Birk Inhibitor, SBBI) prolonged the larval and pupal period of second instar larvae (64–72 h) of *B. cucurbitae*. Punithavalli and Jebamalaimary^32^ too had reported prolonged development of sugarcane borer *Chilo infuscatellus* fed with PI extracted from *Erianthus arundinaceus*. The significant delay observed in larval and total development period in the present study might be due to the deficiency of essential amino acids required for the normal growth and inhibition of trypsin which is a major digestive peptidase^33^. Machado *et al*.^16^ had tested *Acacia polyphylla* seeds (AcKI) containing proteinaceous inhibitors (Kunitz-type inhibitor) against *Anagasta kuehniella* (polyphagous pest) and observed reduction in development, survival and enzymatic activity of *A. kuehniella* larvae.

The activity of trypsin and chymotrypsin decreased at LC_50_ concentration of the SMTI. A previous study reported inhibition in trypsin and chymotrypsin activity in *B. cucurbitae* larvae by the action of peptidase inhibitor extracted from peas^30^. Silva *et al*.^34^ observed that *Adenanthera pavonina* trypsin inhibitor (ApTI) inhibited trypsin and chymotrypsin by 87% and 63%, respectively. The result in this study also revealed that at higher concentrations, the activity of trypsin is more affected by SMTI as compared to that of chymotrypsin suggesting trypsin is an important enzyme in the larval gut. Ferreira *et al*.^35^ found that recombinant PI from *Bauhinia bauhinioides* (kallikrein inhibitor) inhibited the midgut peptidases of *Nasutitermes corniger* workers and soldiers, thus reducing their digestive capacity and affecting the growth of the insect. Saikhedkar *et al*.^36^ reported reduction in expression of HaTry7 (*H. armigera* Trypsin 7) and HaChy4 (*H. armigera* Chymotrypsin 4) gene when *H. armigera* larvae were fed on bicyclic peptide derived from plant Pin-II type PI. Inhibitory activities of peptidase inhibitors from chick pea and black gram against trypsin-like enzymes have also been reported in the larvae of *H. armigera* and *S. litura*, respectively^37,38^. The down regulation of protein digestion in *B. cucurbitae* larvae could be because of the inhibitory action of PIs on insect’s midgut peptidases^39^. The inhibition of digestive peptidase indicates interaction of PIs with the active site of the enzyme, interfering with its digestive function^33^. These findings suggest the interference of SMTI with insect’s metabolism.

This study also evaluated the mRNA expression level of different genes present in melon fruit fly larvae when fed on SMTI incorporated diet. In a previous study it was documented that the herbivorous insects regulate their digestive functions according to diet so that they can evade the inhibitory effect of dietary peptidase inhibitor^40^. The mRNA level of trypsin and chymotrypsin was down-regulated in second instar larvae of melon fruit fly fed on artificial diet incorporated with SMTI. The primary enzymes associated with digestive mechanisms in dipterans are trypsin and chymotrypsin^41^. The inhibition of these enzymes leads to unavailability of essential amino acids causing physiological stress^42^. The mRNA expression level for Alkaline phophatase (AP) increased after SMTI consumption by the larvae, resulting in weight loss and reduced reproductive strength of the insect^43^. AP has been postulated to play a significant role in binding toxins and mediating their insertion into the membrane thus helping in their absorption in the insect^44–46^. SMTI also changed the mRNA expression level of some genes associated with detoxification and antioxidant pathways. The intake of SMTI incorporated artificial diet led to enhanced mRNA expression level of esterases and GST. Insect’s detoxification enzymes are mainly recognized for their role in defending against harmful compounds and maintaining the body’s normal physiological functions^47,48^. The mRNA expression level of both catalase and SOD was also up regulated which suggested metabolic stress in the insect. Catalase and SOD are crucial enzymes that assist an organism to deal with the body’s metabolic stress. SOD helps to maintain natural physiological stability by developing the first line of defense toward different toxic chemicals including plant compounds^49^. Catalase eliminates the hazardous hydrogen peroxide generated by the SOD action^50^.

Borate *et al*.^51^ revealed that anti-microbial activity is also possessed by certain PIs, suggesting that such PIs might be part of proteins related to defensive strategy that offer some protection against fungal and bacterial infections. Previous study^52^ had reported that *Abelmoschus moschatus* trypsin inhibitor from seeds displayed strong antibacterial activity towards *E. coli, Streptococcus pneumoniae, Staphylococcus aureus, Proteus vulgaris, Bacillus subtilis, Bacillus cereus* and moderate activity towards *Pseudomonas syringae, Klebsiella pneumoniae, Streptococcus pyogenes* and *Pseudomonas aeruginosa*. However, it also affected different fungal species, *Candida tropicalis, Candida albicans, Candida glabrata, Asperigillus flavus* and *Asperigillus niger*. Nabi *et al*.^17^ reported that serine PIs purified from the *Sophora japonica* seeds exhibited anti bacterial activity against *Bacillus subtilis, Staphylococcus aureus* and *Streptococcus pneumonia* whereas it didn’t show any inhibitory activity against *E. coli, Klebsiella pneumonia, Saccharomyces cerevisiae* and *Candida albicans*.

## Material and Methods

### Purification of trypsin inhibitor from *S. mukorossi* seeds

The seeds of *S. mukorossi* belonging to the Sapoteaceae family were obtained from Palampur, Himachal Pradesh, India. The endosperms of mature dry seeds of *S. mukorossi* were collected by removing the hard cover using pestle and mortar. To remove any bacterial or fungal contamination, the endosperms were washed with 0.01% mercury chloride and then with double distilled water. The treated endosperms were air dried and fine-ground flour was obtained by crushing them using liquid nitrogen. This flour was then defatted twice by stirring in acetone and hexane (1:1) mixture for 3 h at 4 °C. The defatted seed flour was then air dried and solubilised in 0.01 M sodium phosphate buffer (pH 7.6) by constant stirring for 3 h at 4 °C. Subsequently, the seed suspension was centrifuged at 5000 rpm for 20 min at 4 °C and the pellet was discarded. The supernatant obtained was termed as Crude extract (CE). The ammonium sulphate precipitation of the crude extract was done using different saturation ranges: 0–80% (F1), 20–80% (F2), 40–80% (F3), and 60–80% (F4). The precipitate from each saturation range was centrifuged at 10,000 rpm at 4 °C for 30 min. Each precipitate was then re-suspended in sodium phosphate buffer of 0.01 M (pH 7.6) and dialyzed on a 12 kDa cut off pore membrane for 24 h against the same buffer. The dialyzed sample with maximum trypsin inhibitory activity was subjected to DEAE-Cellulose column (1.5 × 16 cm) chromatography pre equilibrated with sodium phosphate buffer (0.01 M, pH 7.6). To remove the impurities from the column, it was washed with the same buffer and further eluted at a flow rate of 0.5 ml/min with increasing concentrations of NaCl (0.1–0.5 M) dissolved in sodium phosphate buffer (0.01 M, pH 7.6). Trypsin inhibitory activity of the collected fractions (1 ml/fraction) was checked and the fractions with maximum activity were pooled. The collected sample was dialyzed overnight at 4 °C with sodium phosphate buffer (0.01 M, pH 7.6) and further concentrated using amicon filters (Millipore).

The concentrated sample was further purified using CNBr-activated Trypsin-Sepharose affinity column (Sigma Aldrich, Germany). The column was eluted with KCl (2 mM, pH 2.0) for further studies. The fractions possessing maximum trypsin inhibitory activity were pooled and designated as *S. mukorossi* trypsin inhibitor (SMTI). The same SMTI was analyzed using a Reverse Phase-High Performance Liquid Chromatography (Shimadzu RP-HPLC system, LC-60AD) column by employing binary elution system (0–60% acetonitrile and 0.1% aqueous trifluoroacetic acid) at a flow rate of 3 ml/min for 15 min at 30 °C. The elutions were monitored at 220 nm for the detection of protein.

### Protein concentration and Trypsin inhibition assay

Protein quantification was done by the Bradford method using bovine serum albumin (BSA) as a standard^53^. A previously described method was used to determine the inhibition in residual hydrolytic activity of bovine trypsin against BApNA (N α- benzoyl-DL-arginine-p-nitroanilide)^54^. SMTI was incubated with 20 μL trypsin (1 mg/ml in 0.05 M Tris-HCl, pH-8.2) at 37 °C for 10 min. Then, 100 μL of BApNA (prepared in 0.1 mM Dimethyl sulfoxide) was added to the reaction mixture and the change in absorbance was recorded continuously at 405 nm at an interval time of 1 min in a microplate reader (Model 680XR Plate reader (Bio-Rad Lab. Ltd)). One trypsin inhibitor activity unit (TIU) was defined as reduction of 0.01 units of absorbance at 410 nm.

### SDS-PAGE

The homogeneity of SMTI was analyzed on a 12% resolving gel by SDS-PAGE following Laemmli’s method^55^. The molecular weight of the purified SMTI was determined by comparing with the corresponding relative movement of pre stained benchmark protein ladder (Invitrogen).

### Optimal pH, optimal temperature and stability

Optimum pH for SMTI was determined by peptidase inhibitor assay at a range of pH varying from 6 to 11. Substrate casein (1%) was prepared in different buffers of different pH which included phosphate buffers (pH 6–8), Tris – HCl (pH 8–9) and carbonate – bicarbonate buffer (pH 9–11). The stability of SMTI over a range of pH was checked after incubating SMTI in buffers of different pH (6–11) for 30 min, at 4 °C. After incubation, 1 ml of the sample was assayed for trypsin inhibitory activity.

The thermal stability of SMTI was evaluated according to Dias *et al*.^56^. SMTI (1 mg ml^−1^) was dissolved in 1x PBS buffer, pH 7.5, and 100 μL aliquots were incubated in a water bath at different temperatures (10, 20, 30, 40, 50, 60, 70, 80, 90, and 100 °C) for 30 min. Before testing the residual trypsin inhibitory activity, the samples were cooled to room temperature (25 ± 2 °C).

### Effect of metal ions, detergents and organic solvents on peptidase inhibitor activity

Effect of various metal ions (Ca^2+^, Co^2+^, Cu^2+^, Fe^3+^, Zn^2+^, Mg^2+^, Ni^2+^, Mn^2+^) on activity of SMTI was evaluated using 1 mM, 5 mM, 25 mM and 50 mM final concentrations in the reaction mixture. The SMTI equilibrated with different concentrations of above listed metal ions were incubated for 30 min at 25 °C, and then assayed for trypsin inhibitor activity.

Effect of various non-ionic and ionic surfactants such as Triton X-100, SDS, Tween-80, CTAB, and Urea on SMTI activity was determined by incubating SMTI for 30 min with each surfactant at different concentrations 0.1%, 0.5%, 1.0% and 2.0%. Effect of various solvents namely acetone, acetonitrile, benzene, chloroform, DMSO, ethanol, ethylacetate, hexane, methanol and petroleum ether on SMTI activity was determined by incubating SMTI with different concentrations (10%, 20%, 30% and 40%) of these solvents for 30 min at 25 °C.

### IC_50_, IC_90_, Kinetic parameters and Casein hydrolysis test

The SMTI concentration which reduces 50% and 90% of the trypsin activity (IC_50_ and IC_90_) was determined using linear regression as described in previous section^50^ using different SMTI concentrations (0.5 µM to 5.0 µM). The kinetic measurements of SMTI were done following the method of Dias *et al*.^56^. The different concentrations of SMTI (0.5 µM to 5 µM) were incubated with 20 µl of trypsin (1 mg/ml in 0.05 M Tris HCl, pH 8.2) for 10 min at 37 °C. The reaction was initiated by adding 100 μl of different concentrations (0.5 mM to 5 mM) of BApNA. The absorbance change was continuously monitored in a microplate reader (Model 680XR Plate reader (Bio-Rad Lab. Ltd)) for 10 min at 405 nm at an interval time of 1 min. A Line weaver Burk plot was plotted (using Graphpad Prism 8) to calculate Km and V_max_ whereas Ki was calculated using Dixon plot by the intersection of the lines at the x-axis, corresponding to the substrate concentrations (0.5–5.0 µM). The inhibitory activity of SMTI was also examined by simple casein hydrolysis test using agar plates. The agar plates were prepared by mixing the separately autoclaved casein (2%) and bacteriological agar (2%). About 30 µl of trypsin (100 µg/ml) was mixed with each concentration of SMTI (0–100 µg/ml) and the solution was poured into each well made in casein agar plates. The well without SMTI was considered as control and the assays were done in triplicate under controlled conditions.

### Insect assays

#### Laboratory Rearing of Melon Fruit Fly

The adults of melon fruit fly were reared on artificial and natural diet in insect culture room /B.O.D. of the laboratory under controlled conditions of temperature (25 ± 2 °C), relative humidity (70–80%) and photoperiod (10 L:14D).

#### Larval Growth Index (LGI) and Total Growth Index (TGI)

The effect of purified SMTI on second (64–72 h old) instar larvae of melon fruit fly was studied following the method of Srivastava^57^. Melon fruit fly adults deposited eggs on pumpkin pieces placed in wire mesh boxes. The pumpkin pieces (Charged) were then transferred to controlled conditions for egg hatching. Larvae were collected at 64–72 h stage in culture vials containing SMTI incorporated artificial diet (0.2 µg/ml, 1 µg/ml, 5 µg/ml, 25 µg/ml, 125 µg/ml, 625 µg/ml and control (water)). Evaluation of different parameters viz. larval period, total development period, percent pupation and percent adult emergence was done daily. There were six replicates for each concentration with 15 larvae in each replication. Larval Growth Index (LGI) and Total Growth Index (TGI) were calculated by using formula given as^58^.

LGI = Percent Pupation/Larval Period (in days).

TGI = Percentage Emergence/Total Development Period (in days).

### Enzyme assay

LC_50_ was calculated for carrying out the enzyme assays. The second instar larvae of *B. cucurbitae* were fed with LC_50_ concentration of SMTI incorporated in artificial diet. The trypsin and chymotrypsin was extracted and isolated by the method described by Christellar *et al*.^59,60^. Actively feeding larvae were removed in distilled water and the larval homogenate was prepared in 0.15 M NaCl followed by centrifugation at 13500 rpm for 10 min at 4 °C. The supernatant was collected and stored at −20 °C for further analysis. For assay about 50 μl of extract was added to 50 μl of buffer in 96-well Elisa plate and incubated for 10 min at 37 °C. The reaction was started by adding 100 μl of 1 mM of BApNA, mixed in Dimethyl Sulfoxide (DMSO). The reaction was monitored at 405 nm using a microplate reader (Model 680XR Plate reader (Bio-Rad Lab. Ltd)) and residual trypsin inhibitory activity was calculated. Blanks were run in each case by replacing extract with distilled water.

### Gene expression analysis

The relative expression of different enzymes related genes in the second instar larvae of melon fruit fly was measured using quantitative RT-PCR. To extract the total RNA using Trizol method (Invitrogen), the 2^nd^ instar larvae of melon fruit fly fed with LC_50_ concentration of SMTI were collected at 24 h, 48 h and 72 h intervals. Agarose gel electrophoresis (1%) and Nanodrop spectrophotometer were used to check the quality and concentration of total RNA. The iScript cDNA synthesis kit of Biorad was used to synthesize cDNA from 1.0 μg of total RNA. The cDNA was diluted (10 fold) for further use and stored at −20 °C. The mRNA sequences of various genes, used to design primers of genes of interest were obtained from NCBI (Table 4) and actin was used as an internal reference gene. Power SYBR Green PCR Master Mix (Applied Biosystems) with Step One Real Time PCR System (Applied Biosystems) was used to perform the qRT-PCR for different genes. To determine the relative gene expression, 2^−ΔΔCT^ method^61^ was used to calculate the threshold values (*Ct*).

**Table 4:** Primer sequences used for gene expression analysis using qRT-PCR.

Gene Name	Primer sequence	Annealing Temperature (°C)	Product size (bp)	GenBank Accession number
Actin	Forward Primer 5′ CTGCCTCCACCTCCCTGG 3′ Reverse Primer 5′ CGGATATCAACATCGCACTTCA 3′	52	178	JX110854.1
Catalase	Forward Primer 5′ TTTCGAGGTGACGCATGACA 3′ Reverse Primer 5′ ACCAAATCCCAGACACCGTC 3′	52	186	XM_011191851.2
GST	Forward Primer5′ GCGGCATCGAGTACGAAGAT 3′ Reverse Primer 5′ TGTAGATCCTCCCACGGTGT 3′	55	187	XM_011185965.2
Esterase	Forward Primer 5’′GAACGACCAGAGCCTTGGAA 3′ Reverse Primer 5′ GCTTCACCCACAACAAAGCC 3′	55	200	XM_029044934.1
Trypsin	Forward Primer 5′ GCCTTTCTAGGCGACTCTGG 3′ Reverse Primer 5′ CTGAGGCGCCAACTTTAAGC 3′	52	189	XM_011179991.2
Chymotrypsin	Forward Primer 5′ GTGCCGCTCCCTATCAAGTG 3′ Reverse Primer 5′ AGCTCCGGACGGAATGTTAC 3′	52	187	XM_029041388.1
SOD	Forward Primer 5′ ATTATCGGACGCGGTTTGGT 3′ Reverse Primer 5′ GCCAGCACGAAGGCAAATAG 3′	54	194	XM_011194429.2
AP	Forward Primer 5′ TGTTATCTTGGGTGGCGGAC 3′ Reverse Primer 5′ ACCCATCACGTGTGTAGCAG 3′	55	193	XM_011178676.2

GST- Glutathione S-transferase; SOD- superoxide dismutase; AP- alkaline phosphatase.

### Antibacterial activity

The purified SMTI’s antibacterial activity was evaluated using disc diffusion assay against different bacterial strains (*B. thuringiensis, E. coli, P. aeruginosa* and *M. smegmatis)*. Different concentrations of SMTI were soaked in the sterile paper discs (6 mm diameter) and dried in air under sterile conditions. About 100 μL bacterial suspension was spread on LB (Luria Bertani) agar plates after growing the bacterial strains for 24 h. SMTI soaked disks were placed on agar plates and incubated at 37 °C overnight. The discs soaked with different antibiotics (Rifampin-100 µg, Ampicillin-100 µg, Carbenicillin-100 µg, and Kanamycin-50 µg) and sterile distilled water were used as positive and negative control, respectively. The minimum inhibitory concentration (MIC) for different bacterial strains was measured by comparing the zone of inhibition produced by SMTI concentration to that of positive control.

### Statistical analysis

All analyses were reported as means with the standard error. For each set of results, analysis of variance (ANOVA) was applied, followed by the Dunnett or Tukey test.

### Ethical approval and consent to participate

This article does not contain any studies involving human participants or animals performed by any of the authors.

## Conclusion

Our findings suggest that SMTI has potential to impair the growth and development of *B. cucurbitae* larvae by altering its physiological and biochemical processes and can be explored for the development of resistant plants by plant breeders. It is also important to point out that delaying or interrupting the insect life cycle, such as that of *B. cucurbitae*, is an important strategy to mitigate their population without directly killing them, thereby helping in reducing the economic losses caused by phytophagous insect pests. In addition present findings contribute to a better understanding of the anti microbial and insecticidal potential of plant peptidase inhibitors and especially suggest that *S. mukorossi* is a potential source of bioactive proteins. Therefore, SMTI can be employed as an important tool for pest control as it has potential to protect plants against insect pests as well as bacterial pathogens.

## Supplementary information

Supplementary information

## Data Availability

All data generated or analyzed during this study are included in this published article and its additional files.
