# Supplementary material for: Exploration of anti-insect potential of trypsin inhibitor purified from seeds of Sapindus mukorossi against Bactrocera cucurbitae
Source: Sci Rep. 2019 Nov 19;9:17025. doi: 10.1038/s41598-019-53495-6 (PMC6863899; doi:10.1038/s41598-019-53495-6)

Exploration of anti-insect potential of trypsin inhibitor purified from seeds of *Sapindus mukorossi* against *Bactrocera cucurbitae*

Samiksha[1], Drishtant Singh[2], Anup Kumar Kesavan[2] and Satwinder Kaur Sohal[*1]

[1]Department of Zoology, Guru Nanak Dev University Amritsar, Punjab, 143005

[2]Department of Molecular Biology and Biochemistry, Guru Nanak Dev University Amritsar, Punjab, 143005

*Corresponding Author: satudhillon@hotmail.com

Tel.: +91-0183 2258802-09EXTN3398

Fig. S1. LC_{50} concentration of SMTI.

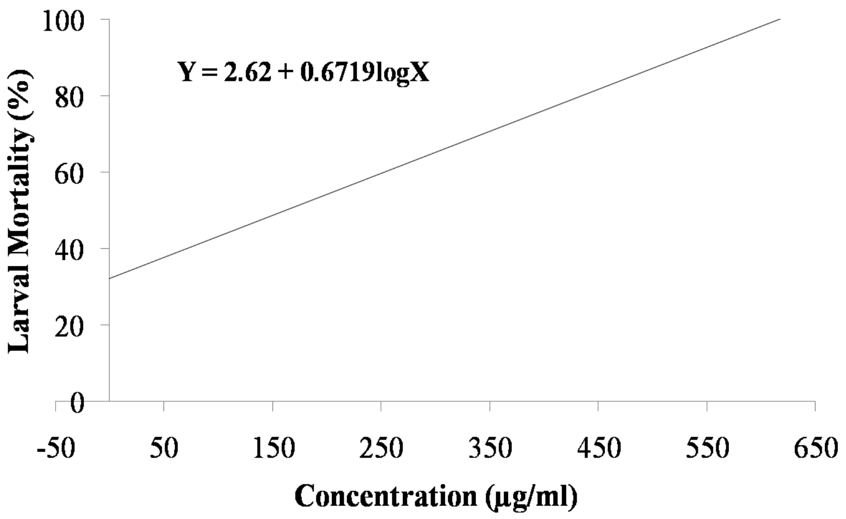

Fig. S2. Antibacterial activity of SMTI **(A)** ***E.coli*** (**B**) ***P. aeruginosa*** **(C)** ***B. thuringiensis***

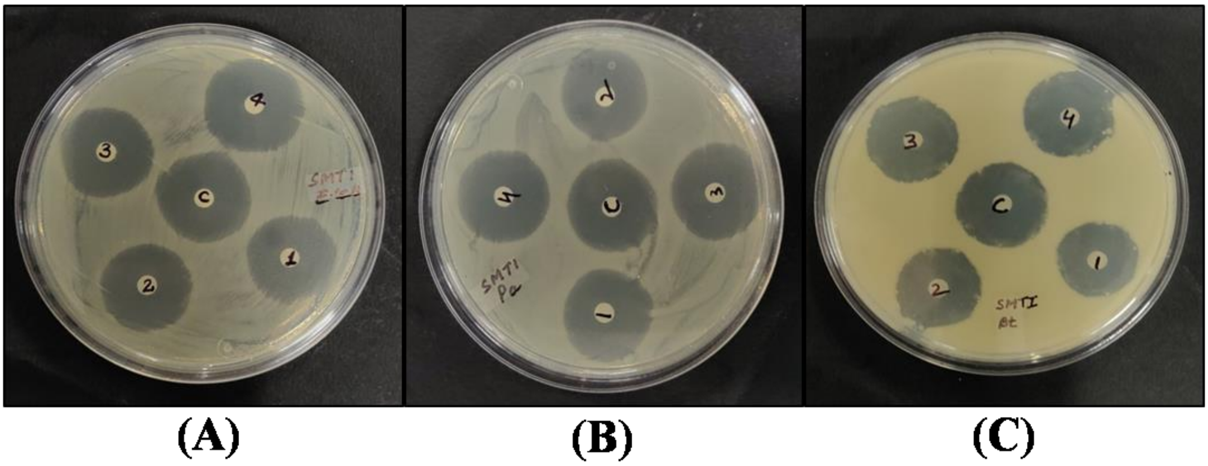

Supplement: Supplementary file 1 — Supplementary information [file 41598_2019_53495_MOESM1_ESM.pdf]
